# Ability of vaccine strain induced antibodies to neutralize field isolates of caliciviruses from Swedish cats

**DOI:** 10.1186/s13028-015-0178-z

**Published:** 2015-12-12

**Authors:** Jonas Johansson Wensman, Ayman Samman, Anna Lindhe, Jean-Christophe Thibault, Louise Treiberg Berndtsson, Margaret J. Hosie

**Affiliations:** Department of Clinical Sciences, Swedish University of Agricultural Sciences, Uppsala, Sweden; MRC-University of Glasgow Centre for Virus Research, Glasgow, UK; Department of Virology, Immune Biology and Parasitology, National Veterinary Institute, Uppsala, Sweden; Global Technical Services, Merial, Lyon, France

**Keywords:** Feline calicivirus, Vaccine, Virus neutralization, Feline upper respiratory tract disease

## Abstract

**Background:**

Feline calicivirus (FCV) is a common cause of upper respiratory tract disease in cats worldwide. Its characteristically high mutation rate leads to escape from the humoral immune response induced by natural infection and/or vaccination and consequently vaccines are not always effective against field isolates. Thus, there is a need to continuously investigate the ability of FCV vaccine strain-induced antibodies to neutralize field isolates.

**Methods:**

Seventy-eight field isolates of FCV isolated during the years 2008–2012 from Swedish cats displaying clinical signs of upper respiratory tract disease were examined in this study. The field isolates were tested for cross-neutralization using a panel of eight anti-sera raised in four pairs of cats following infection with four vaccine strains (F9, 255, G1 and 431).

**Results:**

The anti-sera raised against F9 and 255 neutralised 20.5 and 11.5 %, and 47.4 and 64.1 % of field isolates tested, respectively. The anti-sera against the more recently introduced vaccine strains G1 and 431 neutralized 33.3 and 70.5 % and 69.2 and 89.7 %, respectively. Dual vaccine strains displayed a higher cross-neutralization.

**Conclusions:**

This study confirms previous observations that more recently introduced vaccine strains induce antibodies with a higher neutralizing capacity compared to vaccine strains that have been used extensively over a long period of time. This study also suggests that dual FCV vaccine strains might neutralize more field isolates compared to single vaccine strains. Vaccine strains should ideally be selected based on updated knowledge on the antigenic properties of field isolates in the local setting, and there is thus a need for continuously studying the evolution of FCV together with the neutralizing capacity of vaccine strain induced antibodies against field isolates at a national and/or regional level.

**Electronic supplementary material:**

The online version of this article (doi:10.1186/s13028-015-0178-z) contains supplementary material, which is available to authorized users.

## Background

Feline calicivirus (FCV) is one of the primary causes of infectious upper respiratory tract disease in cats, an important disease worldwide [1]. In addition to respiratory signs, FCV is frequently associated with chronic gingivostomatitis, and highly virulent strains have been associated with an acute systemic virulent disease with a high case fatality rate [2, 3].

Feline calicivirus is a single-stranded RNA-virus of positive sense genome with a high genetic and antigenic variability [4–6], associated with escape from the humoral immune response induced by natural infection and/or vaccination.

FCV is distributed world-wide in the cat population and is estimated to be the etiological cause of upper respiratory tract disease in 10–50 % of cases [1]. The prevalence of FCV varies depending on the management and number of cats in a household [7]. In multi-cat households, such as breeding catteries, cat shelters and cat colonies, FCV is more prevalent [8, 9]. In Sweden, 18 % of feline clinical samples submitted for virus isolation to the National Veterinary Institute during the year 2000 tested positive for FCV, while healthy carriers of FCV in Swedish catteries were estimated at 2.6 % [10].

Several commercial vaccines consisting of various vaccine strains are available, but due to the high mutation rate of FCV, vaccines are not always efficacious [11–13]. There is therefore a need to investigate and follow the ability of vaccine strain induced antibodies to neutralize field isolates of FCV, in order to evaluate and provide updated advice on vaccination strategies at local and/or regional levels.

In a previous study, the cross-neutralization of FCV field strains isolated in the UK by vaccine strain induced antibodies was examined [14]. Here, we used a similar approach to investigate whether comparable findings would apply in a different European country, namely Sweden.

## Methods

### FCV field isolates

A panel of 78 field isolates of FCV grown in vitro was selected from samples submitted between October 2008 and February 2012 to the National Veterinary Institute in Sweden. The samples were all collected from cats showing clinical signs compatible with FCV infection, although the clinical signs were not always stated in the referral form (Additional file 1), and FCV diagnosis had been confirmed by transmission electron microscopy (TEM).

### Virus neutralization test

The isolated FCV isolates were expanded following the inoculation of 2 × 10^5^ cell/mL of feline embryo A (FEA) cells [15] in T25 cm^2^ flasks (Corning, NY). Cells were incubated at 37 °C in an atmosphere of 5 % CO_2_. Cultures were observed for evidence of cytopathic effect (CPE) for up to 4 days post-infection. Once CPE became evident, culture fluids were harvested, passed through a 0.45 μm filter Minisart syringe filter (Sartorius, UK), aliquoted and stored at −80 °C prior to titration.

To titrate the virus isolates, fourfold dilutions of the viral stocks (starting at a dilution of 1/500) were prepared and then incubated (in quadruplicate, per dilution per isolate) in 96-well plates (Thermo scientific, NY) with 2 × 10^5^ cells/mL of FEA cells in a total volume of 200 µl at 37 °C for 48 h. As previosuly, the TCID_50_ was calculated as the reciprocal of the final dilution where CPE was evident in at least 50 % of the wells [14].

Virus isolates were tested for cross-neutralization using a panel of eight anti-sera, raised in four pairs of cats infected once by the oronasal route with 1 mL of each viral inoculum containing 10^6^ TCID_50_ of FCV strain F9, 255, G1 or 431 (one pair of cats was infected with each strain). Serial dilutions of anti-sera (threefold, starting from a 1/5 dilution) were incubated with 100 TCID_50_ of each of the 78 field isolates in quadruplicate in 96-well plates in a total volume of 100 µl for 2 h at 37 °C. Next, 100 µl of FEA cells were added at a density of 2 × 10^5^ cells/mL and the plates were incubated for 48 h. The neutralization titre was calculated as the reciprocal of the highest serum dilution where cells were free from CPE in at least two of four replicate wells.

## Results and discussion

### Virus neutralization of FCV field isolates

The FCV field isolates were analysed in a neutralization test using eight antisera raised against four commercially available FCV vaccine strains (G1, 431, 255 and F9). The same panel of paired anti-sera as had been used by Addie and co-workers [14] was used in this study, with the exception of the anti-sera raised against FCV G1. Fifteen of the field isolates were tested in parallel with the batch of anti-sera previously used [14] as well as the new pair of anti-sera raised against FCV G1, with similar results (Additional file 2). This comparison validated the use of the new batch of anti-sera, which was subsequently used to test all field isolates in this study. The paired anti-sera raised against vaccine strains 255 and F9, which have been used in vaccines for several decades, neutralized 47.4 and 64.1 % (strain 255) or 20.5 and 11.5 % (strain F9) of the field isolates with titres ≥5 (Table 1). The more recently introduced vaccine strains G1 and 431 [16] neutralized 33.3 and 70.5 % (strain G1) or 69.2 and 89.7 % (strain 431) of the field isolates with titres ≥5. In this study, similar differences in neutralizing capacity between paired anti-sera were observed, as had previously been recorded [14] (Table 1), consistent with inter-cat variation in humoral immune responses to FCV infection. As shown in Table 1, strain F9 neutralized few field isolates at titers greater than 45. Only one field isolate (isolated from cat no. 6 displaying upper respiratory signs; Additional files 1 and 2) was not neutralized by any of the anti-sera, and another field isolate (isolated from cat no. 75 with ulcerative stomatitis; Additional files 1 and 2) was neutralized at a titer of five by only one of the anti-sera raised against strain G1 (S2).

**Table 1:** Results of virus neutralization tests

Titres	S1; G1	S2; G1	S3; 431	S4; 431	S5; 255	S6; 255	S7; F9	S8; F9
<5	52 (67)	35 (45)	24 (31)	8 (10)	41 (53)	28 (36)	62 (79)	69 (88)
5–15	20 (26)	22 (28)	39 (50)	32 (41)	28 (36)	19 (24)	12 (15)	7 (9)
45–1215	6 (8)	21 (27)	15 (19)	38 (49)	9 (12)	31 (40)	4 (5)	2 (3)

Antisera raised against four feline calicivirus vaccine strains (G1, 431, 255 and F9) were used in the neutralization tests of 78 Swedish field isolates. The number of field isolates neutralized by respective antiserum is indicated, with the proportion of neutralized strains shown as a percentage within parenthesis

These results are in accordance with previous reports [11, 14, 17], indicating the poor ability of antibodies against FCV vaccine strain F9 to neutralize current field isolates. The reduced capacity of field isolate neutralization is driven by the high mutation rate of FCV to escape the host immune response leading to emergence of escape mutants [6, 18], whether the immunity is induced by vaccination and/or natural infection. The likelihood of reduced protection increases when a vaccine strain has been extensively used for a long period of time. Antibodies raised against the more recently introduced vaccine strains (G1 and 431) demonstrated better neutralization of field isolates compared to those raised against strain F9 (Table 1); however, we observed no marked difference between antibodies raised against these strains compared to FCV vaccine strain 255. To our knowledge, vaccine strain 255 has not been extensively used in Sweden, although it was introduced a few decades ago; thus, it is likely that this strain has not contributed to the immune pressure driving FCV mutation.

### Combining two vaccine strains increases the likelihood of neutralization

Currently, the vaccines registered and in use in Sweden contain either one single vaccine strain (either 255 or F9) or a combination of two vaccine strains (G1 and 431). Therefore, we compared the neutralizing capacity of the anti-sera raised against these vaccine strains as they are presented in current vaccine preparations. Only 10 % of the field isolates were neutralized by both paired anti-sera raised against strain F9, 44 % were neutralized by both paired anti-sera raised against strain 255, whereas 73 % of the field isolates were neutralized by combinations of the anti-sera against strains G1 and 431, where at least one of the paired anti-sera always was neutralizing (Table 2). These findings indicate that by combining two vaccine strains, the likelihood of neutralization of the field isolates increases. It is of interest to note that, by combining strains 431 and 255 in a putative vaccine preparation, an even broader neutralizing capacity (60/78; 77 %) would have been seen against this panel of field isolates (data not shown). This effect could be the result of the greater phylogenetic distance in amino acid sequence between isolates 431 and 255, compared to isolates 431 and G1 [16].

**Table 2:** Virus neutralization tests interpreted by combining data from antisera raised against vaccine strains as currently presented in registered vaccine preparations

G1 and 431					255			F9		
G1 and 431 always neg.	G1 and 431 possibly neg.	G1 neg. or possibly neg. and 431 always pos.	G1 always pos. and 431 neg. or possibly neg.	G1 and 431 always pos.	Always neg.	Possibly neg.	Always pos.	Always neg.	Possibly neg.	Always pos.
2	19	34	4	19	25	19	34	61	9	8
3 %	24 %	44 %	5 %	24 %	32 %	24 %	44 %	78 %	12 %	10 %

Feline calicivirus (FCV) vaccines registered and used in Sweden either contain F9 or 255 only, or a combination of G1 and 431 vaccine strains. The cutoff for a positive neutralizing titer was set to ≥5. The result “possibly negative” indicates that one of the paired neutralizing sera was negative, while the other gave a positive (≥ 5) neutralization titer

The combination of two antigenically distant vaccine strains has previously been proposed in order to achieve higher cross-neutralizing capacity [14]. When we examined data from a previous study of field isolates from the UK, a similar comparison (combining the results obtained for antisera raised against two vaccine strains available in one of the current vaccine preparations) gave comparable results; 72 % of the field isolates were neutralized by antibodies raised against strains G1 or 431, whereas antibodies raised against either strain 255 or F9 neutralized 34 or 15 %, respectively [14]. The use of dual vaccine strains has also been shown to be more effective in the protection against the virulent systemic (VS-) FCV strains [19], although the vaccine strains were not comparable to those used in the present study. Furthermore, there was no suggestion of VS-FCV strains being included among the field isolates used here (Additional file 1).

There is an ongoing discussion that FCV isolated from cats displaying clinical signs of chronic FCV infection are more often vaccine resistant [20, 21], and thus not as often neutralized by anti-sera raised against vaccine strains. Since this study was designed to investigate the capacity of different vaccine strains to neutralize field isolates from Swedish cats, it is not possible to draw any conclusions about the relation between clinical signs and virus neutralization based on the clinical information stated by the veterinary clinicians at submission of samples.

## Conclusions

This study confirms previous observations that antibodies raised against more recently introduced vaccine FCV strains, or vaccine strains used less widely, cross neutralize a higher proportion of circulating field isolates than antibodies raised against strains that have been used in vaccines extensively for a long time. Moreover, we demonstrated higher cross-neutralization of field isolates when considering neutralization data for all of the antisera raised against two strains included in a dual FCV vaccine, compared to the neutralization observed by the antisera raised against single vaccine strains. Vaccine strains should ideally be selected based on updated knowledge on the antigenic properties of field isolates in the local setting, and there is thus a need to continuously study the evolution of FCV and the neutralizing capacity of vaccine strain-induced antibodies against field isolates at a national and/or regional level.

## Additional files

10.1186/s13028-015-0178-z Clinical information about cats from which FCV was isolated. The table provides clinical information about the cats from which the field isolates originate, including year of isolation, breed, age, and clinical signs as stated on the referal form. DSH = domestic shorthair, ESH = European shorthair, y = years, m = months, w = weeks, - denotes information not available.
10.1186/s13028-015-0178-z Virus neutralization data. The Excel file lists the titers obtained in neutralization tests using vaccine strain induced anti-sera against the field isolates. Field isolates 64–78 were tested in parallel with the previously used batch of anti-sera (indicated S1§ and S2§) [14], and the new batch of anti-sera, with similar results.

## References

[1] Sykes JE (2014). Pediatric feline upper respiratory disease. Vet Clin North Am Small Anim Pract.

[2] Pedersen NC, Elliott JB, Glasgow A, Poland A, Keel K (2000). An isolated epizootic of hemorrhagic-like fever in cats caused by a novel and highly virulent strain of feline calicivirus. Vet Microbiol.

[3] Coyne KP, Jones BR, Kipar A, Chantrey J, Porter CJ, Barber PJ (2006). Lethal outbreak of disease associated with feline calicivirus infection in cats. Vet Rec.

[4] Coyne KP, Edwards D, Radford AD, Cripps P, Jones D, Wood JL (2007). Longitudinal molecular epidemiological analysis of feline calicivirus infection in an animal shelter: a model for investigating calicivirus transmission within high-density, high-turnover populations. J Clin Microbiol.

[5] Coyne KP, Christley RM, Pybus OG, Dawson S, Gaskell RM, Radford AD (2012). Large-scale spatial and temporal genetic diversity of feline calicivirus. J Virol.

[6] Radford AD, Dawson S, Ryvar R, Coyne K, Johnson DR, Cox MB (2003). High genetic diversity of the immunodominant region of the feline calicivirus capsid gene in endemically infected cat colonies. Virus Genes.

[7] Radford AD, Coyne KP, Dawson S, Porter CJ, Gaskell RM (2007). Feline calicivirus. Vet Res.

[8] Polak KC, Levy JK, Crawford PC, Leutenegger CM, Moriello KA (2014). Infectious diseases in large-scale cat hoarding investigations. Vet J.

[9] Hellard E, Fouchet D, Santin-Janin H, Tarin B, Badol V, Coupier C (2011). When cats’ ways of life interact with their viruses: a study in 15 natural populations of owned and unowned cats (Felis silvestris catus). Prev Vet Med.

[10] Holst BS, Berndtsson LT, Englund L (2005). Isolation of feline herpesvirus-1 and feline calicivirus from healthy cats in Swedish breeding catteries. J Feline Med Surg.

[11] Lauritzen A, Jarrett O, Sabara M (1997). Serological analysis of feline calicivirus isolates from the United States and United Kingdom. Vet Microbiol.

[12] Radford AD, Bennett M, McArdle F, Dawson S, Turner PC, Glenn MA (1997). The use of sequence analysis of a feline calicivirus (FCV) hypervariable region in the epidemiological investigation of FCV related disease and vaccine failures. Vaccine.

[13] Radford AD, Dawson S, Coyne KP, Porter CJ, Gaskell RM (2006). The challenge for the next generation of feline calicivirus vaccines. Vet Microbiol.

[14] Addie D, Poulet H, Golder MC, McDonald M, Brunet S, Thibault JC (2008). Ability of antibodies to two new caliciviral vaccine strains to neutralise feline calicivirus isolates from the UK. Vet Rec.

[15] Jarrett O, Laird HM, Hay D (1973). Determinants of the host range of feline leukaemia viruses. J Gen Virol.

[16] Poulet H, Jas D, Lemeter C, Coupier C, Brunet S (2008). Efficacy of a bivalent inactivated non-adjuvanted feline calicivirus vaccine: relation between in vitro cross-neutralization and heterologous protection in vivo. Vaccine.

[17] Rinaldo D, Foti M, Bottari T, Fisichella V, Buonavoglia D (2008). Feline calicivirus strains isolated in Italy. Pol J Vet Sci.

[18] Radford AD, Turner PC, Bennett M, McArdle F, Dawson S, Glenn MA (1998). Quasispecies evolution of a hypervariable region of the feline calicivirus capsid gene in cell culture and in persistently infected cats. J Gen Virol.

[19] Huang C, Hess J, Gill M, Hustead D (2010). A dual-strain feline calicivirus vaccine stimulates broader cross-neutralization antibodies than a single-strain vaccine and lessens clinical signs in vaccinated cats when challenged with a homologous feline calicivirus strain associated with virulent systemic disease. J Feline Med Surg.

[20] Porter CJ, Radford AD, Gaskell RM, Ryvar R, Coyne KP, Pinchbeck GL (2008). Comparison of the ability of feline calicivirus (FCV) vaccines to neutralise a panel of current UK FCV isolates. J Feline Med Surg.

[21] Poulet H, Brunet S, Soulier M, Leroy V, Goutebroze S, Chappuis G (2000). Comparison between acute oral/respiratory and chronic stomatitis/gingivitis isolates of feline calicivirus: pathogenicity, antigenic profile and cross-neutralisation studies. Arch Virol.

