# Erratum to: Ability of vaccine strain induced antibodies to neutralize field isolates of caliciviruses from Swedish cats

**DOI:** 10.1186/s13028-016-0194-7

**Published:** 2016-02-17

**Authors:** Jonas Johansson Wensman, Ayman Samman, Anna Lindhe, Jean-Christophe Thibault, Louise Treiberg Berndtsson, Margaret J. Hosie

**Affiliations:** 1 Department of Clinical Sciences, Swedish University of Agricultural Sciences, Uppsala, Sweden; 2 MRC-University of Glasgow Centre for Virus Research, Glasgow, UK; 3 Department of Virology, Immune Biology and Parasitology, National Veterinary Institute, Uppsala, Sweden; 4 Global Technical Services, Merial, Lyon, France

## Erratum to: Acta Vet Scand (2015) 57:86 DOI 10.1186/s13028-015-0178-z

Unfortunately, the original version of this article [1] contained an error. In the Abstract, Results and Discussion it reads:

The more recently introduced vaccine strains G1 and 431 [16] neutralized 33.3 and 70.5 % (strain G1) or 69.2 and 89.7 % (strain 431) of the field isolates with titres ≥5.

However it should be:

The more recently introduced vaccine strains G1 and 431 [16] neutralized 33.3 and 55.1 % (strain G1) or 69.2 and 89.7 % (strain 431) of the field isolates with titres ≥5.
