# Supplementary material for: Ability of vaccine strain induced antibodies to neutralize field isolates of caliciviruses from Swedish cats
Source: Acta Vet Scand. 2015 Dec 12;57:86. doi: 10.1186/s13028-015-0178-z (PMC4676829; doi:10.1186/s13028-015-0178-z)
Supplement: Supplementary file 1 — 10.1186/s13028-015-0178-z Clinical information about cats from which FCV was isolated. The table provides clinical information about the cats from which the field isolates originate, including year of isolation, breed, age, and clinical signs as stated on the referal form. DSH = domestic shorthair, ESH = European shorthair, y = years, m = months, w = weeks, - denotes information not available. [file 13028_2015_178_MOESM1_ESM.docx]

**Additional file 1 – Clinical information about cats from which FCV was isolated**

| **Field isolate ID/**  **year of sampling** | **Breed / Age** | **Clinical signs** |
| --- | --- | --- |
| 1 / 2008 | DSH / 6 y | - |
| 2 / 2008 | Cornish Rex / 6 w | Rhinitis |
| 3 / 2008 | ESH / 4 y | Chronic gingivostomatits |
| 4/ 2008 | Maine Coon / 1 y | - |
| 5 / 2009 | Ragdoll / 1 y | Erosions in oral mucosa |
| 6 /2009 | Siamese / 1 y | Upper respiratory signs |
| 7 / 2009 | DSH / 6 y | Gingivostomatitis |
| 8 / 2009 | DSH / 3 m | Lip and tongue ulcers |
| 9 / 2009 | DSH / 9 y | Oral ulcers |
| 10 / 2009 | Abyssinian / 9 m | Chronic hyperplastic gingivitis |
| 11 / 2009 | DSH / 5 y | Dental problems |
| 12 / 2009 | DSH / 9 m | - |
| 13 / 2009 | DSH / - | Ulcerations in oral mucosa, pyrexia |
| 14 / 2009 | DSH / 1 y | Chronic gingivitis |
| 15 / 2009 | - / - | Ulcerative stomatitis |
| 16 / 2009 | - / 14 y | Gingivostomatitis |
| 17 / 2009 | Norwegian Forest / 1 y | Chronic gingivitis, head tilt |
| 18 / 2009 | Persian / 1 y | Chronic gingivitis |
| 19 / 2009 | DSH / 6 m | Stomatitis, sneezing |
| 20 / 2009 | Abyssinian / 1 y | Chronic gingivostomatitis |
| 21 / 2009 | Maine Coon / 10 m | - |
| 22 / 2009 | DSH / 6 m | Proliferative gingivitis |
| 23 / 2009 | Persian / 2 y | - |
| 24 /2009 | Maine Coon / 1.5 y | Gingivitis with plaque |
| 25 / 2009 | Norwegian Forest / 8 m | Gingivostomatits |
| 26 / 2010 | DSH / 6 m | Gingivitis with blisters |
| 27 / 2010 | Oriental Shorthair /5 m | - |
| 28 / 2010 | Maine Coon / 4 m | - |
| 29 / 2010 | DSH /13 y | - |
| 30 / 2010 | Norwegian Forest/10 y | Chronic gingivostomatitis |
| 31 / 2010 | DSH / 10 y | Ulcerations in tongue and oral mucosa |
| 32 / 2010 | Maine Coon / 1 y | - |
| 33 / 2010 | Maine Coon / 2 y | - |
| 34 / 2010 | DSH / 8 m | Hyperplasic ulcerative gingivitis |
| 35 / 2010 | DSH / 2 y | Hyperplasic gingivitis, FORL |
| 36 / 2010 | Siamese / 13 y | Gingivitis, FORL, tongue erosions |
| 37 / 2010 | Cornish Rex / 8 y | Chronic gingivostomatitis |
| 38 / 2010 | Maine Coon / 8 m | Chronic gingivitis |
| 39 / 2010 | Maine Coon / 4 y | Hypersalivation, oral pain, pyrexia |
| 40 / 2010 | DSH / 4 y | Gingivostomatitis |
| 41 / 2010 | Maine Coon / 1 y | - |
| 42 / 2010 | DSH / 9 y | Chronic stomatitis |
| 43 / 2010 | Persian / 6 m | - |
| 44 / 2010 | Ragdoll / 2 y | Chronic gingivitis |
| 45 / 2010 | Maine Coon / 1 y | Upper respiratory signs, follicles in pharynx, tonsillitis |
| 46 / 2010 | Maine Coon / 1 y | Upper respiratory signs |
| 47 / 2010 | DSH / 4 y | - |
| 48 / 2010 | Maine Coon / 6 m | Gingivostomatitis, tracheitis, rhinitis |
| 49 / 2010 | Maine Coon / 7 m | Hyperaemic gingivitis |
| 50 / 2010 | Maine Coon / 9 m | - |
| 51 / 2010 | DSH / 2 y | Chronic stomatitis |
| 52 / 2010 | Maine Coon / 4 m | Nasal congestion, stomatitis |
| 53 / 2010 | DSH / 6 y | Excessie erosive gingivostomatitis |
| 54 / 2011 | Norwegian Forest /9 m | Tongue erosions, hypersalivation, enlarged lymph nodes |
| 55 / 2011 | Maine Coon / 6 m | Excessive gingivostomatitis |
| 56 / 2011 | Maine Coon / 1 y | - |
| 57 / 2011 | DSH / 3 y | Chronic gingivostomatitis |
| 58 / 2011 | Devon Rex / - | Excessive gingivitis |
| 59 / 2011 | Persian / 9 y | Purulent ocular and nasal discharges, stomatitis |
| 60 / 2011 | Siamese / 5-6 w | Upper respiratory signs, tongue erosions, pyrexia |
| 61 / 2011 | Siamese / 1 y | Upper respiratory signs, conjunctivitis, tongue erosions, pyrexia |
| 62 / 2011 | DSH / 13 y | Gingivostomatitis |
| 63 / 2011 | Cornish Rex / 6 y | Oral cavity signs |
| 64 / 2011 | DSH / 3 y | Excessive gingivitis, oral pain |
| 65 / 2011 | Persian / 5 y | Ulcerative stomatitis, anorexia |
| 66 / 2011 | Norwegian Forest/10 y | - |
| 67 / 2011 | British Shorthair / 5 y | Epulis, gingivitis |
| 68 / 2011 | DSH / 8 y | Erosive vesicular stomatitis |
| 69 / 2011 | Persian / 10 m | Stomatitis, nasal and ocular discharges |
| 70 / 2011 | Egyptian Mau / 2.5 y | Excessive gingivostomatitis, tongue ulcers |
| 71 / 2011 | Norwegian Forest/1.5 y | - |
| 72 / 2011 | DSH / 3.5 y | Excessive gingivostomatitis |
| 73 / 2011 | Ragdoll / 1 y | Ulcers in gingiva and oral mucosa |
| 74 / 2011 | DSH / - | Conjunctivitis, perforated cornea ulcus |
| 75 / 2011 | Siamese / 2 y | Ulcerative stomatitis |
| 76 / 2011 | Maine Coon / 12 w | Tongue ulcers, halitosis |
| 77 / 2012 | Ragdoll / 6 m | Sneezing, nasal discharges, halitosis, gingivitis |
| 78 / 2012 | Norwegian Forest / 8 w | Lameness, pyrexia, blisters at tongue and palate |
